# Deep Brain Stimulation of the Subthalamic Nucleus Does Not Affect the Decrease of Decision Threshold during the Choice Process When There Is No Conflict, Time Pressure, or Reward

**DOI:** 10.1162/jocn_a_01252

**Published:** 2018-06-01

**Authors:** Friederike Leimbach, Dejan Georgiev, Vladimir Litvak, Chrystalina Antoniades, Patricia Limousin, Marjan Jahanshahi, Rafal Bogacz

**Affiliations:** University College London Institute of Neurology; University College London Institute of Neurology; University College London Institute of Neurology; University of Oxford; University College London Institute of Neurology; 6 University College London Institute of Neurology; 7 University of Electronic Science and Technology of China; University of Oxford

## Abstract

During a decision process, the evidence supporting alternative options is integrated over time, and the choice is made when the accumulated evidence for one of the options reaches a decision threshold. Humans and animals have an ability to control the decision threshold, that is, the amount of evidence that needs to be gathered to commit to a choice, and it has been proposed that the subthalamic nucleus (STN) is important for this control. Recent behavioral and neurophysiological data suggest that, in some circumstances, the decision threshold decreases with time during choice trials, allowing overcoming of indecision during difficult choices. Here we asked whether this within-trial decrease of the decision threshold is mediated by the STN and if it is affected by disrupting information processing in the STN through deep brain stimulation (DBS). We assessed 13 patients with Parkinson disease receiving bilateral STN DBS six or more months after the surgery, 11 age-matched controls, and 12 young healthy controls. All participants completed a series of decision trials, in which the evidence was presented in discrete time points, which allowed more direct estimation of the decision threshold. The participants differed widely in the slope of their decision threshold, ranging from constant threshold within a trial to steeply decreasing. However, the slope of the decision threshold did not depend on whether STN DBS was switched on or off and did not differ between the patients and controls. Furthermore, there was no difference in accuracy and RT between the patients in the on and off stimulation conditions and healthy controls. Previous studies that have reported modulation of the decision threshold by STN DBS or unilateral subthalamotomy in Parkinson disease have involved either fast decision-making under conflict or time pressure or in anticipation of high reward. Our findings suggest that, in the absence of reward, decision conflict, or time pressure for decision-making, the STN does not play a critical role in modulating the within-trial decrease of decision thresholds during the choice process.

## INTRODUCTION

Converging behavioral and neurophysiological data suggest that during decision-making between two options, the evidence favoring one alternative over another is integrated over time (Ratcliff & McKoon, 2008; Gold & Shadlen, 2007; Schall, 2001). These data also suggest that the choice is made when the amount of integrated evidence reaches a particular value, referred to as a *decision threshold*. Humans and animals have an ability to adjust the decision threshold according to instructions to be fast or accurate or according to task demands (Wickelgren, 1977). It has been suggested that the adjustments of decision threshold rely on the BG circuit, and several possible mechanisms have been proposed (Bogacz, Wagenmakers, Forstmann, & Nieuwenhuis, 2010; Forstmann et al., 2008; Furman & Wang, 2008; Frank, Scheres, & Sherman, 2007). One of these theories suggests that the threshold is controlled by the activity level in the subthalamic nucleus (STN), which through its widespread connections can control the excitability in the output of the BG and thus gate decisions (Frank, Scheres, et al., 2007). This theory has been supported by reports that deep brain stimulation (DBS) of the STN affected the extent to which patients with Parkinson disease (PD) were able to vary their decision threshold according to speed versus accuracy instructions on a motion discrimination task (Pote et al., 2016; Green et al., 2013), and following unilateral subthalamotomy, patients with PD failed to show context-dependent modulation of decision thresholds on a conditional stop signal task (Obeso et al., 2014). Moreover, the patterns of neural activity in the STN differed depending on whether the patients with PD were asked to be fast or accurate on a motion discrimination task (Herz et al., 2017). Furthermore, in a task-switching paradigm, a high threshold after a cue predicting a switch was associated with a higher activity in the STN (Mansfield, Karayanidis, Jamadar, Heathcote, & Forstmann, 2011). However, the theory of threshold control by the STN is not aligned with the observation that the ability to vary decision threshold was not predicted by the amount of structural connectivity between the cortex and the STN but rather between the cortex and the striatum (Forstmann et al., 2010).

Recently, it has been hotly debated whether the decision threshold remains constant or decreases within the choice process. On difficult trials on which both options receive similar amounts of evidence, such decrease would prevent excessively long deliberation (Cisek, Puskas, & El-Murr, 2009). Neural signatures of decreasing thresholds have been observed in firing rates of neurons in areas involved in the choice process. Namely, it was shown that, on average, the activity of neural populations selective for all options increases with time, suggesting that they receive increasing “urgency” inputs, which with time drive all neural populations closer to the response threshold (Churchland, Kiani, & Shadlen, 2008). However, it has proven more challenging to establish if the decrease in decision threshold can be inferred from behavioral data. On the one hand, models with decreasing thresholds described better the distribution of RTs produced by monkeys in the motion discrimination tasks (Ditterich, 2006) and by humans when responses had to be made before a deadline (Murphy, Boonstra, & Nieuwenhuis, 2016). On the other hand, comprehensive studies showed that multiple data sets from classic decision-making paradigms from humans are better explained by models with constant rather than decreasing thresholds (Voskuilen, Ratcliff, & Smith, 2016; Hawkins, Forstmann, Wagenmakers, Ratcliff, & Brown, 2015). These studies had to infer the slope of the boundary indirectly by fitting computational models to behavioral data, as in classic decision-making paradigms information is presented continuously so it is difficult to estimate what evidence the participant could have integrated at the moment of choice. To infer the decision boundary more directly, a recent study employed an expanded judgment paradigm in which information is presented in discrete steps, so that it is known what total evidence was presented on each trial before the participant made the choice (Malhotra, Leslie, Ludwig, & Bogacz, 2017). This study demonstrated that humans indeed employ decreasing decision thresholds in certain conditions.

Previous studies suggested that DBS disrupts the functions in which the STN is thought to be involved in the healthy brain, such as preventing prepotent responses when they are inappropriate (Georgiev, Dirnberger, Wilkinson, Limousin, & Jahanshahi, 2016; Wylie et al., 2010; Thobois et al., 2007; Hershey et al., 2004; Jahanshahi et al., 2000; for a review, see (Jahanshahi, Obeso, Baunez, Alegre, & Krack, 2015). Specifically, Frank and colleagues (2007) proposed that the STN is involved in changing the height of the threshold within a trial. They studied the effects of STN stimulation on decision-making between alternatives that were previously associated with different reward probabilities. Results suggested that STN stimulation impaired decision-making in high-conflict situations in which both of the presented options had been associated with high reward probability. This was reflected in the failure of patients with PD to slow down in high-conflict trials when STN DBS was on, unlike when their DBS was off or the unoperated PD and healthy controls (Frank, Samanta, Moustafa, & Sherman, 2007). Similar findings were reported with the same task by Cavanagh et al. (2011), who additionally recorded scalp EEG and showed that STN stimulation reversed the normal increase in theta-band activity over the medial pFC, which is usually associated with raising the decision threshold for high-conflict trials, thus suggesting that STN DBS interferes with the normal ability of the STN to react to decision conflict by modulating the decision threshold (Cavanagh et al., 2011). In addition, STN DBS has been reported to disrupt slowing of RTs on conflicting or difficult trials in perceptual decision-making tasks (Green et al., 2013; Coulthard et al., 2012) and to result in fast but errorful responses under speed pressure (Pote et al., 2016).

In addition to the role of the STN in increasing the threshold within a trial, it has also been suggested that the decrease in threshold during a choice trial reflects reduced inhibition from the STN (Ratcliff & Frank, 2012). Here, we asked if the STN is causally involved in reducing the decision threshold with time within a trial. To address this question, we studied the decision-making of patients with PD with DBS applied to the STN. We asked patients with PD to perform an expanded judgment task with STN DBS switched on or off and investigated whether STN DBS reduces the slope of decrease of decision threshold.

## METHODS

### Participants

Thirteen patients (11 men) with a clinical diagnosis of PD based on the U.K. Brain Bank criteria were assessed (Hughes, Daniel, Kilford, & Lees, 1992). All patients had bilateral STN DBS for 6 months or longer. The mean age was 61.6 (*SD* = 10.04, range 42–73). To control for possible practice and age effects, 23 neurologically healthy participants with no history of psychiatric illness, head injury, or alcohol or drug abuse were recruited, of whom 11 were matched in age with the patient group (6 men) and 12 were younger (8 men). The mean age of the age-matched group was 66.0 (*SD* = 12.28, range 45–82), and the mean age of the young group was 29.3 (*SD* = 4.65, range 24–35). Patients and older controls were matched in age (*p* > .05). The demographic and clinical information for the samples are presented in Table 1. The stimulation parameters for each patient are presented in Table 2. All patients were assessed on medication.

**Table 1.** Demographic and Clinical Information for the Three Groups

*Group*	*PD*	*Age Matched*	*Young*
Age, years	61.6 (10.04)	66.0 (12.28)	29.3 (4.65)
Sex			
Male	11	6	8
Female	2	5	4
Years of education	13.8 (2.65)	16.5 (2.07)	19.4 (2.72)
MMSE	28.7 (2.02)	29.3 (0.90)	29.7 (0.89)
Digit Span	18.2 (3.74)	18.2 (3.16)	20.4 (3.75)
BDI	8.8 (3.63)	3.7 (3.07)	2.3 (2.18)
SAS	15.0 (5.45)	10.5 (5.28)	5.9 (3.18)
UPDRS motor part			
On	18.1 (6.85)		
Off	37.0 (9.87)		

Mean and standard deviation values (in brackets) for age, years of education, Mini Mental State Examination (MMSE), digit span, Beck Depression Inventory (BDI), Starkstein Apathy Scale (SAS), and Unified Parkinson Disease Rating Scale-Part III (UPDRS).

**Table 2.** Stimulation Parameters for the Left and Right Electrodes Implanted in the STN for the 13 Patients with PD

*Patient*	*Left STN*				*Right STN*			
	*Active Contact*	*Voltage (V)*	*Frequency (Hz)*	*Pulse Width (μsec)*	*Active Contact*	*Voltage (V)*	*Frequency (Hz)*	*Pulse Width (μsec)*
1	−2, −3	4.0	60	100	−11	3.1	60	100
2	−0	2.3	60	80	−8	3.0	60	80
3	−2	2.5	160	60	−9	2.3	160	60
4	−1	3.4	80	60	−5	3.3	80	60
5	−1	1.6	130	60	−9	1.8	130	60
6	−1, −2	1.1	160	60	−8	1.1	160	60
7	−1	2.5	130	60	−9	2.5	130	60
8	−1	1.65	80	60	−8	2.2	80	60
9	−0	2.8	80	60	−10	2.8	80	60
10	−2	1.9	125	60	−9	1.4	125	60
11	−1	4.2	80	60	−5	3.2	80	60
12	−2, −3	3.9	60	80	−5	3.5	60	80
13	−1	4.1	125	60	−8	3.9	125	60
Mean		2.77	102.31	66.15		2.62	102.31	66.15

The Mini-Mental State Examination, the Beck Depression Inventory, and the Starkstein Apathy Scale were administered to screen for dementia, clinical depression, and apathy. None of the patients had cognitive impairment, clinical depression, or apathy.

### Task

**Figure 1.**
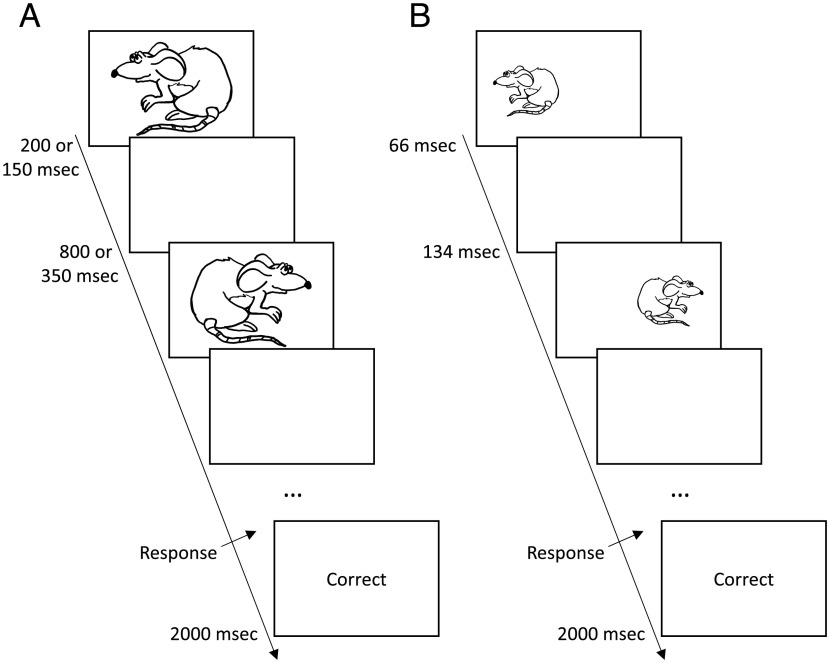
Timeline of a single trial. (A) Slow and medium rate conditions. In the slow condition, the stimulus was presented for 200 msec followed by 800 msec of blank screen, whereas in the medium condition, the stimulus was presented for 150 msec and blank screen for 350 msec. (B) In the fast condition, the stimulus was presented for 66 msec followed by a blank screen for 134 msec.

Participants were asked to perform a computerized expanded judgment task developed in MATLAB. During each trial, participants were instructed to predict if a mouse would run left or right. Each trial included multiple presentations of stimuli of a mouse facing either to the left or to the right (Figure 1A). The participants were told that “The mouse is more likely to look in the direction it will run, but sometimes it looks in the other direction.” The stimuli were selected stochastically such that the probability of mouse looking in the “correct” direction was .7. The same randomly pregenerated sequences of stimuli were used for all participants. On each trial, the stimuli were presented until a response was indicated by the participant by pressing the appropriate right or left buttons of a response box with their right or left index finger, respectively. After each response, participants were given visual feedback (correct or incorrect).

The patients performed the task in three conditions differing in the rate of presentation of the stimuli, namely the stimuli were presented every 200, 500, or 1000 msec. The fastest rate (200 msec) was the same as in a previous study of the slope of the decision threshold (Malhotra et al., 2017). In the medium and slow rate conditions, stimuli were presented in the center of the screen (Figure 1A), whereas in the fast rate condition, the mice looking left were presented on the left side of the screen, and vice versa, to make the direction easier to identify within a short period (Figure 1B).

Each condition started with practice trials. During piloting, we found that some participants had a tendency to respond after seeing only one stimulus. To illustrate the benefit of integrating evidence, in the initial 10 practice trials the participants were asked to wait for a “Go” cue on the screen before pressing a button; the different numbers of stimuli were presented on each trial. In the next 20 practice trials (and in the main experiment), the stimuli were presented sequentially until the participant pressed a response button. Subsequently, participants completed two experimental blocks of 50 trials, separated by a break. At the end of each block, they were provided with the percentage of correct responses.

### Design

Patients completed the task with three different rates (200, 500, and 1000 msec) on three separate days. On each day, they were assessed with their stimulators being switched on and with stimulators switched off. The order of the stimulation condition was counterbalanced across patients. The healthy control participants completed the task only with the medium speed rate (500 msec), as we did not expect the rate of stimulus presentation to affect patients and controls in different ways. Control participants were also tested twice to ensure they performed the same number of trials in the medium speed condition as the patients, to control for practice effects.

### Exclusion Criteria

**Figure 2.**
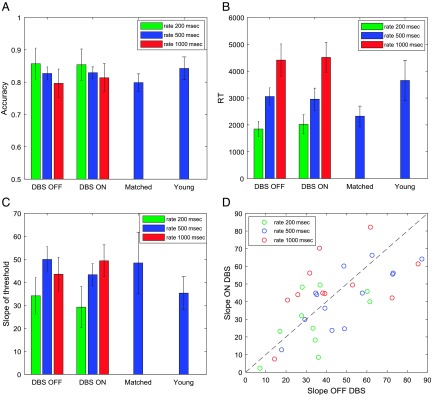
Comparison of behavior across experimental groups and conditions. Different colors correspond to different rate conditions (see key). (A) Objective accuracy defined as the fraction of trials on which “Correct” feedback was given. (B) Mean RT. (C) Slope of decision threshold. In A–C, the error bars show the *SEM*. (D) Distribution of slopes across patients. Each circle corresponds to data from a particular patient in a particular rate condition, and its coordinates are the estimated slopes off and on STN DBS. The dashed line shows the identity line, so that the points below it have a higher slope off STN DBS, whereas points above it have a higher slope on STN DBS. Matched = age-matched healthy controls; Young = young healthy controls.

As the task was relatively easy, participants typically obtained high accuracy (Figure 2A), but a few patients, on some of the study days, performed at chance level. We excluded data from conditions in which the subjective accuracy (defined as the fraction of trials on which response agreed with the majority of stimuli presented on a trial) did not significantly differ from that expected by chance (i.e., when it was below 59.8% corresponding to a nonsignificant *Z* test). This resulted in excluding some of the data from three patients: Patient 1, both DBS conditions with 200-msec rate and both DBS conditions with 1000-msec rate; Patient 4, both DBS conditions with 200-msec rate and both DBS conditions with 1000-msec rate; and Patient 11, both DBS conditions with 200 msec.

### Data Analysis

For each participant and condition, we estimated the slope of decrease of decision threshold using the method developed and tested in a previous study (Malhotra et al., 2017). This method assumes that a state of a decision-maker after seeing *i*th stimulus during the choice process is described by the integrated evidence for the chosen option *x*_*i*_ and time step *t*_*i*_. For example, consider a trial in which a participant observes sequence: left, right, left, left, and then chooses the left response. The integrated evidence increases by 1 each time a stimulus corresponding to the chosen option is presented and decreases by 1 otherwise, resulting in the following sequence: (*t*_0_ = 0, *x*_0_ = 0), (*t*_0_ = 1, *x*_0_ = 1), (*t*_0_ = 2, *x*_0_ = 0), (*t*_0_ = 3, *x*_0_ = 1), (*t*_0_ = 4, *x*_0_ = 2). We looked for a line in the (*t*, *x*) space, such that the states were typically below it before making the decision and above it after the last stimulus. Denoting making a choice by 1 and waiting by 0, we can define a sequence of actions *a*_*i*_, which for our sample trial would be *a*_0_ = 0, *a*_1_ = 0, *a*_2_ = 0, *a*_3_ = 0, *a*_4_ = 1. The probability of an action can be related to the predictor variables using the following logistic regression model,

$$\log\frac{P\left(a_{i}=1\right)}{P\left(a_{i}=0\right)}=\beta_{0}+\beta_{t}t_{i}+\beta_{x}x_{i}$$

In the above equation, β_*t*_ and β_*x*_ are the regression coefficients for time and evidence, respectively, and β_0_ is the intercept. Given the triplet (*x*_*i*_, *t*_*i*_, *a*_*i*_) for each stimulus in each trial, we estimated for each participant and condition the β_*t*_, β_*x*_, and β_0_ that maximized the likelihood of the observed triplets. Next, the slope of the decrease of decision threshold was taken as arctan(β_*t*_/β_*x*_). This slope is equal to 0 if the threshold is constant within a trial and takes positive values if the threshold decreases.

We assessed how the accuracy, RT, and slope of the threshold depended on experimental group and condition. For each of these measures, for the patients we tested how it depended on presentation rate and STN stimulation condition and performed repeated-measures ANOVA with two factors: Rate (200, 500, 1000 msec) and DBS (on vs off). In addition, to compare performance in the medium rate condition between the groups, an ANOVA was performed with one factor: Group (patients with DBS on, patients with DBS off, age-matched controls, young controls).

## RESULTS

### Behavior

Patients had significantly reduced motor symptoms, as measured by the Unified Parkinson Disease Rating Scale-Part III, when the DBS was on in comparison to the condition when it was off (Table 1; *p* < .001, *t* = 7.96, *df* = 12). Some patients received DBS with frequencies above 100 Hz whereas others received DBS with lower frequencies (Table 2), but these two groups did not significantly differ in any behavioral measure reported below, and thus, the analyses of pooled data from both groups are presented.

Figure 2 (A and B) shows the accuracy and RTs of different groups in different conditions of the experimental task. In the medium rate condition, the patients with DBS on or off, elderly and young healthy control groups did not differ significantly in the accuracy (*p* = .70, *F* = 0.48, *df* = 3) or RT (*p* = .34, *F* = 1.15, *df* = 3). The accuracy of the patients did not significantly differ with rate (*p* = .20, *F* = 1.66, *df* = 2) or with the stimulation being on or off (*p* = .74, *F* = 0.11, *df* = 1), and there was no interaction (*p* = .88, *F* = 0.12, *df* = 2). The RT of the patients depended on the rate of the presentation of the stimuli (*p* < .0001, *F* = 39.68, *df* = 2). Thus, the patients responded faster if the stimuli were presented at a faster rate. Nevertheless, the patients did not maintain across rates the average number of stimuli they were viewing before making a choice. For example, the interval between the onset of stimuli is twice as long in the slow (1000 msec) than in the medium condition (500 msec), but the RT in the slow condition is far from being twice as long as in the medium condition. Thus, the patients on average were viewing fewer stimuli before making a choice in the slower condition. However, the RT did not change with the stimulation (*p* = .80, *F* = 0.06, *df* = 1), and there was no interaction (*p* = .87, *F* = 0.13, *df* = 2).

### Slopes of the Decision Threshold

**Figure 3.**
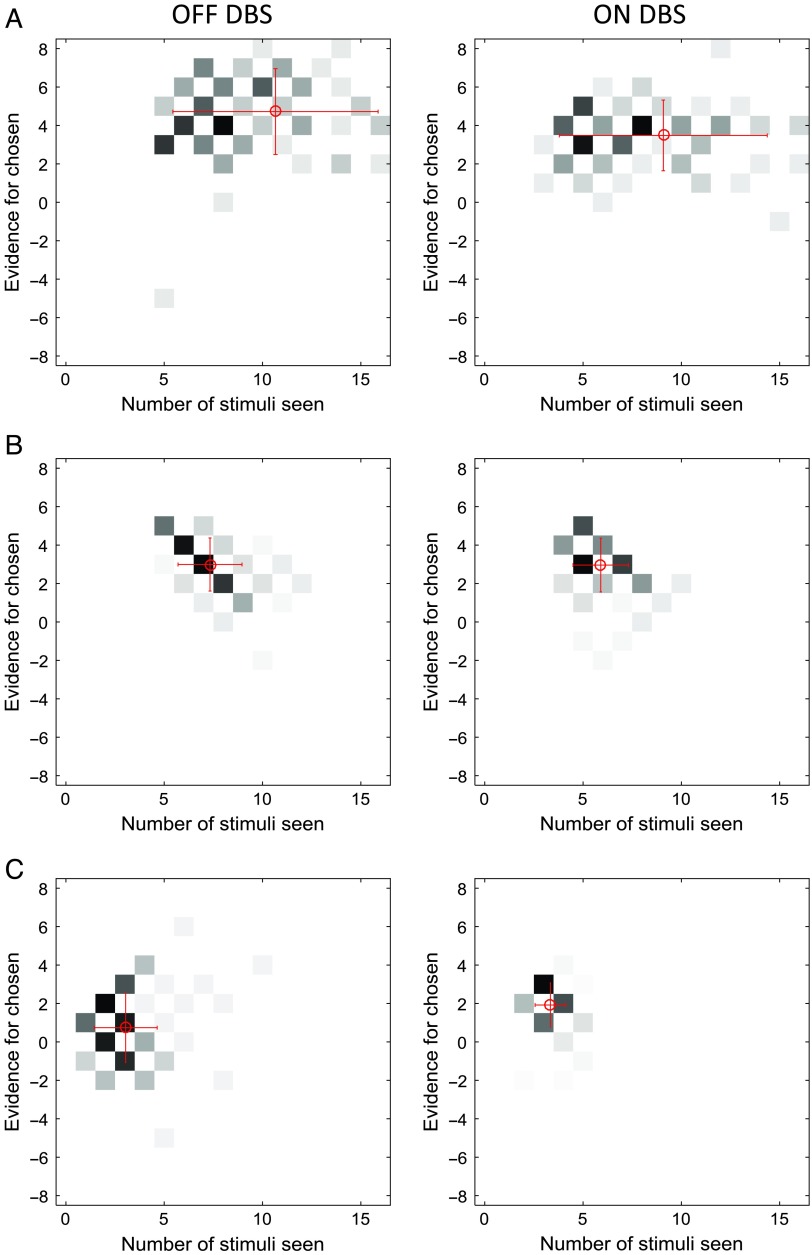
Examples of distributions of combinations of time and evidence at which choices were made. Different panels correspond to different patients. Data in A come from the fast rate condition, whereas data in B and C come from the slow rate condition. Displays in the left column show data from an STN DBS off condition, whereas displays in the right column correspond to STN DBS on condition. In each display, the darkness of a square indicates the number of trials at which the choice was made after seeing a particular number of stimuli (*x* axis) and particular accumulated evidence (*y* axis). The color scale is normalized such that black corresponds to the maximum of a histogram. The coordinates of red circles indicate the mean number of stimuli and evidence, whereas the whiskers indicate standard deviations.

Patients showed a great diversity of slopes of decision threshold as illustrated in Figure 3, which shows at what combinations of time and evidence the choices were made. The patient in Figure 3A tended to respond when the cumulated evidence was in a particular range (around three to seven in the DBS off condition and around two to five in the DBS on condition) irrespective of the time from trial onset. The behavior of this patient is best described by a relatively flat decision threshold (slopes 7° and 2° in the DBS off and on conditions, respectively). By contrast, the patient shown in Figure 3B responded at lower evidence levels as time progressed, and the behavior is best described by decreasing boundaries (slopes 39° and 45° in the off and on conditions). On the other hand, the patient shown in Figure 3C tended to respond after seeing a particular number of stimuli irrespective of the integrated evidence (around two to three stimuli in the DBS off condition and three to four stimuli in the DBS on condition), and this behavior is described by steeply decreasing thresholds (slopes 85° and 61° in the off and on conditions). Although the moment of the response is mostly determined by the number of stimuli seen by this patient, the actual response (left or right) is influenced by the stimuli seen, which allows the patient to achieve an accuracy above chance.

Figure 2D shows the distribution of the slopes of decision threshold across patients. The behavior illustrated in Figure 3A and C corresponds to the lowest and one of the highest slopes, and it can be seen in Figure 2D that most patients had estimated slopes between these extremes. Similar slopes were produced by healthy control participants, and Figure 2C compares the mean slopes across the groups and conditions. In the medium rate condition, the groups did not differ significantly in the slope (*p* = .57, *F* = 0.68, *df* = 3). The slope of the patients' thresholds differed between the rate conditions (*p* = .015, *F* = 4.58, *df* = 2), and it can be noticed in Figure 2D that the points corresponding to the fast rate condition (green circles) tend to have lower slopes of the threshold. There is no systematic difference in the slopes between 500- and 1000-msec rate conditions (Figure 2C) despite the difference in the number of stimuli viewed (Figure 2B), but note that the number of stimuli viewed is not only determined by the slope of the threshold but also by its intercept.

The slope of the threshold did not depend significantly on the DBS condition (*p* = .58, *F* = 0.32, *df* = 1), and there was no interaction (*p* = .27, *F* = 1.35, *df* = 2). Figure 2D does not show any obvious tendency for the points to lie on either side of the identity line. To quantify this further, we compared the coordinates of points in Figure 2D, with a paired Bayesian *t* test, using statistical software JASP 0.8.5.1. The Bayes factor, that is, the ratio of the probability of the null hypothesis (no difference between slopes) over the alternative hypothesis was 4.26. The patients produced similar slopes in the two DBS conditions, and there was a significant correlation between the slopes produced off and on DBS across patients and conditions (*r* = .78, *p* < .0001).

## DISCUSSION

In this study, the estimated slopes of the decision threshold differed significantly as a function of the rate of presentation of the stimuli, with faster rates associated with lower slopes of decision threshold, suggesting that patients adjusted the slope of decision threshold within a trial according to task demands, but the slope was not affected by STN DBS. Decreasing decision thresholds were recently also observed in a task employing a similar expanded judgment paradigm (Malhotra et al., 2017). A steeper decrease of the threshold in a slower rate condition may be connected with a higher mental cost of maintaining the information between the stimuli, which has been proposed to affect the slope of decision boundaries (Drugowitsch, Moreno-Bote, Churchland, Shadlen, & Pouget, 2012).

The lack of the effect of STN DBS on changing the slope of decision threshold within a trial might be related to the differences in the tasks. First, this study did not explicitly introduce any conflict between responses. Unlike the study of Frank and colleagues (2007), where some trials involved the choice between two options previously associated with a high probability of reward, in our study each stimulus provided evidence for one option or another but never for both options. Second, the presented experiment employed an expanded judgment paradigm in which responses were much slower than in previous studies. It is possible that in such slow tasks the behavior is more affected by a process of forcing decisions to move on to the next trial (Drugowitsch et al., 2012) than by a process of slowing down responses in high-conflict situations (Frank, Samanta, et al., 2007). This dominant effect of this forcing process is manifested by extremely steep decision thresholds employed by some participants (Figure 3C). It is possible that the processes of slowing down and speeding up the decision may have different neural bases, as patterns of activity in distinct frequencies have been observed in the STN when patients were asked to be accurate and fast (Herz et al., 2017). Furthermore, STN DBS induced impulsive action reflected as fast and errorful responding and lowering of the decision threshold on the moving dots task was only observed under speed instructions when patients with PD were acting under time pressure (Pote et al., 2016). This is consistent with the proposal that the BG invigorate the decision-making process by providing an urgency signal (Thura & Cisek, 2017). Third, it is possible that, in our expanded judgment task, some participants used explicit strategies, which contributed to the diversity of slopes of decision threshold. This diversity might have masked the effects of DBS. Finally, in the study by Frank and colleagues (2007), decisions were based on previously learned stimulus–action–reward associations, and STN stimulation only produced impulsive behaviors when decision-making in win–win situations. Our study did not involve reward-based decisions, and the function of the STN may be more related to decisions that lead to positive outcomes.

The lack of the effect of DBS may also be connected with the differences in methodology. In the study by Frank and colleagues (2007), the patients were tested on relatively low doses of medication that might have magnified the effects of DBS, whereas in our study, the patients had normal doses of their medications. Furthermore, in previous research patients with STN DBS were tested in two different sessions on different days with their stimulation on or off. The patients were less likely to become fatigued during the sessions. In this investigation, patients were tested on and off stimulation during one session with potential fatigue or “carryover” effects, despite the counterbalancing of the order. Given many differences in tasks and methodology between our and previous studies, a very interesting direction of future work would be to systematically perform a series of experiments, including a repetition of a previous study that showed an effect of DBS, and several conditions each differing in just a single aspect from the repetition. It would allow to identify which features are critical for the effect of DBS on decision-making to be present.

Previous research looking at the acute effects of STN stimulation on decision-making reached contradicting conclusions. Although some authors found deficits with STN stimulation on (Pote et al., 2016; Florin et al., 2013; Green et al., 2013; Coulthard et al., 2012; Cavanagh et al., 2011; Oyama et al., 2011; Rogers et al., 2011; Frank, Samanta, et al., 2007), others reported that decision-making remained stable or even improved with STN stimulation on relative to when the DBS was off (Brandt et al., 2015; Fumagalli et al., 2015; Boller et al., 2014; Torta et al., 2012; van Wouwe et al., 2011). To assess risk-taking and delay aversion, Torta and colleagues (2012) administered the Cambridge Gambling Task and the Quick Delay Questionnaire to assess how STN stimulation affected willingness of patients with PD to respond later to receive bigger rewards. The results suggested that the patients' response delays did not change with STN stimulation (Torta et al., 2012). These findings match our results, considering that we did not find any changes in RT slowing between patients in the on and off stimulation conditions.

In conclusion, our data suggest that stimulation of the STN does not impair the patients' ability to reduce their decision threshold with time within a trial in a relatively slow expanded judgment paradigm. This is in contrast to previous studies suggesting a significant effect of STN DBS on the ability to adjust the decision threshold between trials and on adjusting threshold within a trial in more rapid tasks performed under speed pressure or associated with reward or conflict. Thus, our data raise the possibility that the proposed role of the STN in adjusting thresholds within a trial is specific to rapid decision-making where it is important to slow down the process when conflicting information is present or high reward is at stake.

## Acknowledgments

This research was supported by MRC grant MC_UU_12024/5. The Wellcome Centre for Human Neuroimaging was supported by core funding from the Wellcome Trust (203147/Z/16/Z). The authors thank Ewa Bogacz for drawing the stimuli.

Reprint requests should be sent to Rafal Bogacz, MRC Brain Networks Dynamics Unit, Nuffield Department of Clinical Neuroscience, University of Oxford, John Radcliffe Hospital, Oxford, OX3 9DU, United Kingdom, or via e-mail: rafal.bogacz@ndcn.ox.ac.uk.
